# The Aerodynamic Signature of Running Spiders

**DOI:** 10.1371/journal.pone.0002116

**Published:** 2008-05-07

**Authors:** Jérôme Casas, Thomas Steinmann, Olivier Dangles

**Affiliations:** 1 University of Tours, Institut de Recherches sur la Biologie de l'Insecte, UMR CNRS 6035, 37200 Tours, France; 2 IRD, UR 072, LEGS, UPR 9034, CNRS, 91198 Gif-sur-Yvette, France; 3 Université Paris-Sud 11, 91405 Orsay, France; Smithsonian Institution, United States of America

## Abstract

Many predators display two foraging modes, an ambush strategy and a cruising mode. These foraging strategies have been classically studied in energetic, biomechanical and ecological terms, without considering the role of signals produced by predators and perceived by prey. Wolf spiders are a typical example; they hunt in leaf litter either using an ambush strategy or by moving at high speed, taking over unwary prey. Air flow upstream of running spiders is a source of information for escaping prey, such as crickets and cockroaches. However, air displacement by running arthropods has not been previously examined. Here we show, using digital particle image velocimetry, that running spiders are highly conspicuous aerodynamically, due to substantial air displacement detectable up to several centimetres in front of them. This study explains the bimodal distribution of spider's foraging modes in terms of sensory ecology and is consistent with the escape distances and speeds of cricket prey. These findings may be relevant to the large and diverse array of arthropod prey-predator interactions in leaf litter.

## Introduction

Many predatory species can switch between foraging modes, usually alternating between an ambush and a cruising mode in water, soil or vegetation. Much care has been taken in evolutionary ecology to evaluate the relative advantages of foraging strategies in terms of energetics, biomechanics, success rate and impact on the ecosystem [1]–[7]. However, the relationship between the sensory processes involved in signal production by a predator attacking with one of both strategies and the corresponding signal perception by its escaping prey is unknown for most systems. The outcome of this relationship is likely to play an important role in defining the most appropriate predatory foraging mode. For instance, wolf spiders pursue their cricket prey on the bare soil and in leaf litter using two attack strategies [8]–[10]. Spiders attack prey using either an extremely slow-motion approach, corresponding almost to the ambush strategy, or by running over at relatively high speed (up to 40 cm/s, cruising strategy) [10]. Spiders attack at intermediate speeds much less frequently; biotests using a piston mimicking the attack of a spider showed that a cricket's chances of survival were highest for attacks at intermediate speed (20 cm/s) [10]. Although crickets and many other detritivorous and herbivorous arthropods are sometimes caught unaware by a spider's fast strike, they often escape with fast movements. Information contained in air signals upstream from running spiders can be used by prey in these fast escape reactions. Indeed, crickets, cockroaches and other orthropteroid insects are equipped with air-flow sensors (filiform hairs) at the rear end of their abdomen [11]. They possess many short hairs, serving as acceleration sensors, and fewer long hairs (velocity sensors) on their cerci [12]. These mechanosensors are among the most sensitive sensors in the animal kingdom, with action potentials triggered by less than one tenth the energy of a photon [13]; indeed, the orthropteroid escape system, and in particular fluid flow sensing using filiform hairs, has maintained textbook-example status over many years [14]–[17]. Thus, we hypothesised that spiders use the two different hunting strategies to cope with optimal air-flow detection by crickets. One strategy (ambush) substantially reduces the distance at which the prey can perceive the attack, while the other strategy (cruising) reduces the escape probability by overwhelming the prey sensory capabilities. The high speed ensures that the encounter occurs faster than the escape response.

The aims of this study were therefore: (1) to quantify the air flow in front of a running spider using digital particle imaging velocimetry (DPIV), and (2) to assess these complex flow patterns in the context of attack and escape strategies by predators and prey. Very little is known about air movements upstream from a running arthropod, limiting potential evaluation of the ecological and evolutionary importance of air-flow sensing for many predator-prey interactions. Near-field fluid movement cues are used by many invertebrate species to obtain information about potential predators, prey or mates, in both terrestrial and aquatic ecosystems. In particular, several recent studies have led to greater understanding of the physics of near-field fluid motion in animal locomotion and sensing in open enclosures. Such technological and conceptual advances have opened up the arena for similar studies on running animals [18]–[23].

## Results

**Figure 1:**
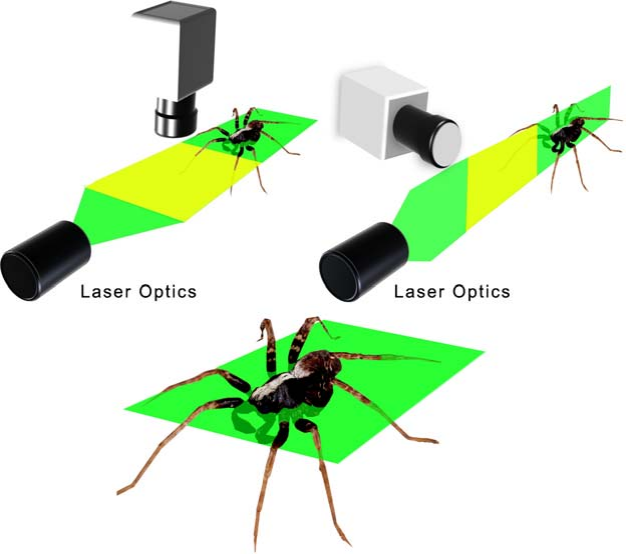
Digital particle image velocity (DPIV) measurements of a running spider. In the horizontal position, the laser light sheet is focussed 3 mm above the floor, at mid-height of the spider, just below the bottom eye row level. The yellow portion represents the camera's field of view. Spiders were gently triggered to run using a stick inserted through a small hole at the entrance of the wind tunnel.

**Figure 2:**
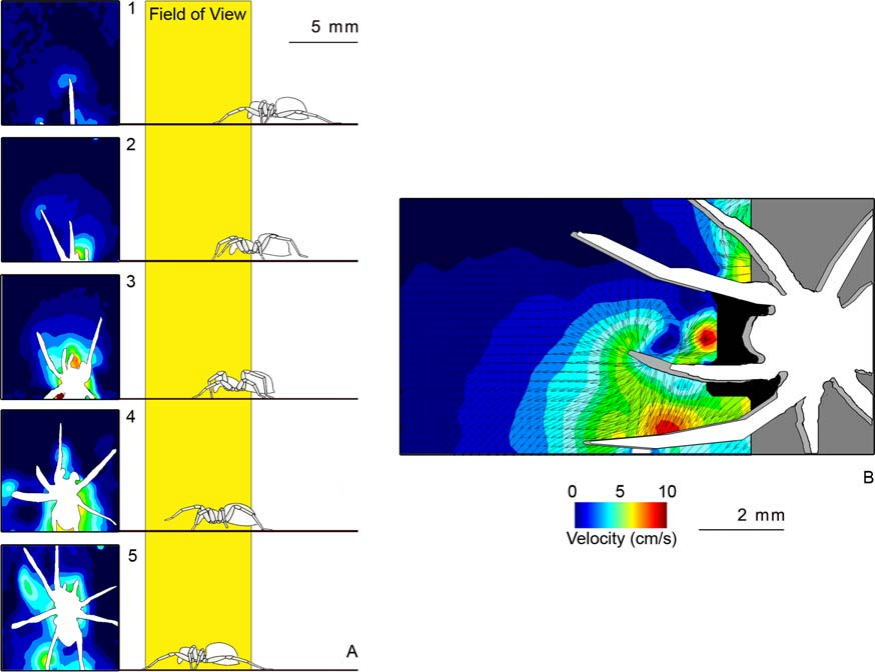
Horizontal flow field and close-up view of the flow around a running spider. The sequence in (A) highlights the pockets of high air-flow velocity created by leg strokes superimposed on the air movements created by the body trunk movement. Neither the tips of the spider's legs, nor their associated flow patterns, are visible as they are located below the light sheet. The time delay between two images is 500 µs; the spider was running at a speed of 5.7 cm/s. The cartoon, adapted from [9], highlights the relative position of legs to body trunk. An overlay of two images (first image in white, second image in grey) of the moving spider, separated by 500 µs, is shown in (B). The zone of flow velocities above the measurable range is in black. The running speed was 10.5 cm/s.

**Figure 3:**
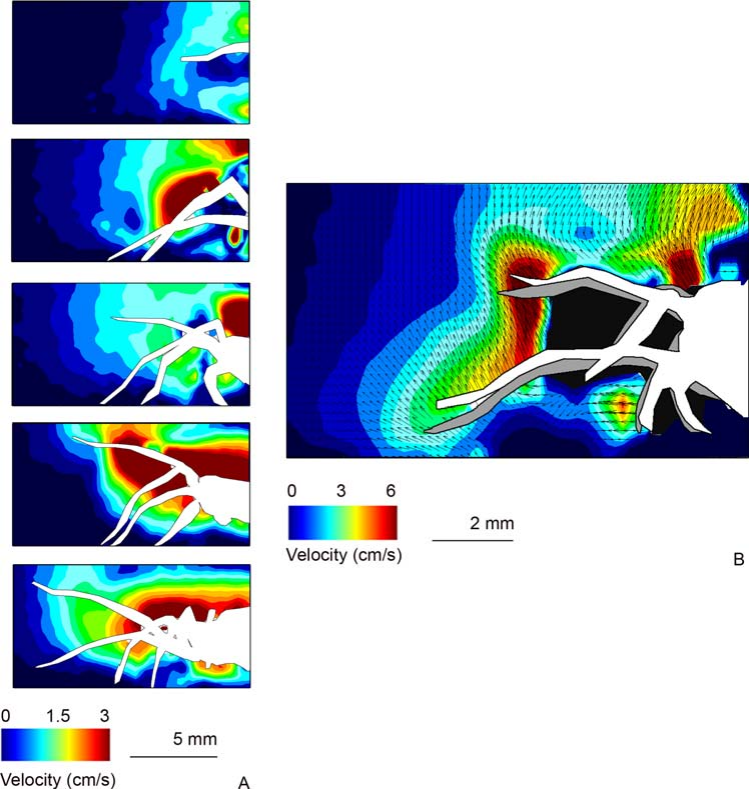
Vertical flow field and close-up view of the flow around a running spider. The sequence in (A) highlights the high air-flow velocity above the spider's body. The time delay between two images is 500 µs; the spider was running at a speed of 3.7 cm/s. An overlay of two images (first image in white, second image in grey) of a moving spider, separated by 500 µs, is shown in (B). The horizontal component of the air flow in the near vicinity of the legs is always directed forward, as front legs do not move back and forth (see cartoon in Figure 2). The running speed was 21 cm/s.

**Figure 4:**
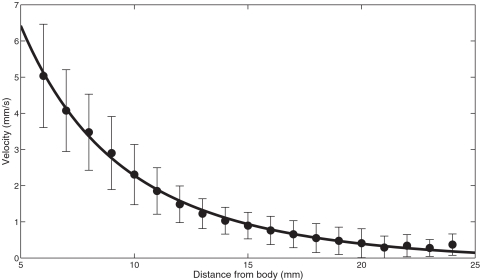
Flow velocity upstream of running spiders. The observed speeds (mean and standard deviation; dots and error bars, respectively), and the fit of the statistical function (Eq. 2) are represented.

We recorded the air flow produced by wolf spiders (*Pardosa* [*lugubris*] sp., most likely P. *lugubris* (Walkenaer)) running in a small wind tunnel (Figure 1). As spiders dislike the intense laser light sheet, we obtained 14 runs from six different individuals with the horizontal set-up, but only two runs with the vertical set-up. These were not used in the following quantitative analysis, but gave useful information on several other qualitative aspects of the flow, described below. The mean velocity of the spiders recorded in the horizontal set-up was 9.44 cm/s (SD = ±5.51; N = 14). This lies within the range of attack speeds observed under unconstrained hunting behaviour [10]. One spider ran at a high speed of 40 cm/s. This was an outlier in the velocity distribution, and so was not used to calculate the mean. Running spiders displaced air in front of and above their body trunk (Figures 2 & 3). Pockets of high velocity produced by moving legs could be distinguished and substantially extended the region of flow influenced by the spider (Figure 2). Front legs still produce a forward air movement when moving downwards, as they do not move back and forth (Figure 3B and cartoon on Figure 2). The air field within the first centimetre upstream from a spider varies considerably from run to run because it is not possible to synchronise the PIV clock with the leg kinematics. Thus, depending on the exact moment of flow field mapping, a leg may or may not have a large effect on the flow in its near vicinity (see cartoon, Figure 2). This also explains the absence of relationship observed between the air velocity at 6 mm away from the spider and the spider's body velocity, and our subsequent decision to pool individual runs for a statistical analysis. The air flow upstream from a running spider declines smoothly with distance (Figure 4); a constrained regression, using the function given in (Eq. 2) and the independently measured mean spider's velocity as a fixed parameter, lead to a good fit over the whole range of distances (R^2^ = 0.80).

## Discussion

The air field upstream from a running spider is disturbed over a large distance of several body lengths. The need for prey to perceive attacking predators from as large a distance as possible, using the minimal amount of energy, means that this information is of biological importance. Indeed, previous experimental studies on the air flow produced by attacking toads shooting out their toungs [24] and independent theoretical studies [25] suggested that cockroaches may recognise the wind signature of a predator by the low frequency components in the far field. The most sensitive hairs of crickets are the longest ones (>1000 microns), working near the thermal noise level [13]. Electrophysiological studies estimate their minimal threshold at V_thresh_ = 30 µm/s. Thus, using the expected flow velocity upstream from a running spider from the fitted model, this threshold should be attained at around 3 cm in front of a spider. This distance, obtained using the observed mean speed, will vary as a function of the speed of the spider. Crickets seem to make full use of this information, with their largest escape distances being 2.4 cm in front of a spider and 2.1 cm in front of a piston device mimicking the kinematics of the attack [10]. This is most impressive, given the time taken for processing information in the abdominal terminal ganglion, the insect brain, and from leg movements [26]. Thus, the cricket's entire escape system, including sensory and locomotive control, is indeed optimised to pick up the slightest air movements by the best sensors.

**Figure 5:**
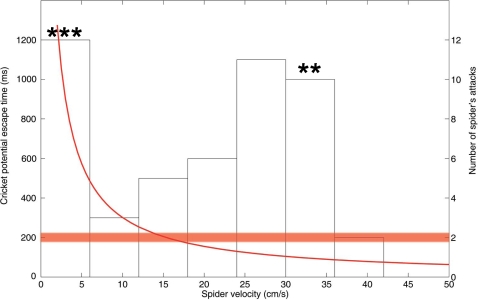
Spider's attack speed and cricket escape time. The potential escape time for a cricket (red line) is expressed as a function of the spider's attack speed. At slow attack speeds, the distance at which crickets can perceive spiders is limiting (ambush strategy), whereas at high hunting speeds, the escape time becomes limiting (cruising strategy). The potential escape time is defined as the time interval between predator perception by a cricket and hit by a spider running at a given speed. It is based on the distance, for a given speed, at which the threshold of 30 µm/s for danger perception is attained [13]. The minimal recorded escape time for crickets is around 0.2 ms (horizontal bar, [26]). The distribution of observed attack speeds and the five successful attacks (stars) were obtained from observations of real attacks, at constant speeds, during cricket-spider interactions [10].

The implications of our results for the foraging modes of spiders are twofold. First, spiders markedly increase their likelihood of successful attacks by launching fast strikes, at the same time decreasing the potential escape time (time between danger perception by a cricket and encounter by a spider) in a non-linear fashion (Figure 5). While low speed movements imply high potential escape times, the distance at which prey can perceive predatory signals is so short that prey are nearly within reach of spiders (ambush strategy). Second, their highest speeds may correspond to the lowest potential prey escape time [26]. Such attack speeds are between 25–35 cm/s, corresponding well with the higher speeds distribution observed during spider-cricket interactions. Higher hunting speeds are seldom observed, as they do not increase the capture rate but are energetically expensive. Thus, our quantification of air flow upstream from a running predator extends the interpretation of the two foraging modes in terms of sensory processes, beyond the classical description in energetic and biomechanical terms. Future studies dedicated to body and leg kinematics should be prioritised, since our understanding of this subject is substantially poorer than that of wing and leg kinematics in insects, and their influence on the upstream flow. The role of acceleration, body posture and height over the substrate [27], [28] as well as the nature of the substrate, aspects which we have neglected here, are also expected to have a major impact on the flow field upstream from the spider.

Many other invertebrate predators, including several other arachnid groups, carabid, cincidelid and staphylinid beetles, hunt prey using the same two strategies as those used by spiders. At the same time, many prey living in litter harbour well-developed cerci bearing filiform hairs triggered by slight air movements. These include primitive and modern insects such as bristletails, firebrats, springtails, cockroaches and crickets; indeed, most prey-spider interactions observed today are the same as they were some 400 million years ago [29], [30]. For example, cockroaches have been extremely successful and thrive in tropical leaf litter despite strong predator pressure. Our findings demonstrate a significant role of the physical information contained in slight air currents in interspecific interactions among terrestrial arthropods and suggest a tight sensory coevolution between both opponents. Lurking predators may mostly hide and wait for their prey, but the final strike produces conspicuous signals that prey exploit for their survival.

## Materials and Methods

### DPIV

Our measurement set-up was composed of a sealed glass box (10×2×2 cm), seeded with 0.2 µm oil particles. Oil particles (Di-Ethyl-Hexyl-Sebacat, 0.5 L, TPAS, Dresden, Germany) were generated using an aerosol generator (ATM 230, ACIL, Chatou, France). The laser (NewWave Research Solo PIV 2, Nd∶YAG, dual pulsed; Dantec Dynamics A/S, Skovlunde, Denmark) illuminated the flow produced by the spider's displacement through glass. The laser sheet (width = 17 mm, thickness at focus point = 50 µm) was operated at low power (3 mJ at 532 nm) to minimise glare. A target area (17×30 mm) was then imaged onto the CCD array of a digital camera (Photron FastCam X1280 PCI 4K) using a Macro Lens (Nikon, AF Nikkor, 60 mm, f : 2.8). The CCD captured separate image frames (1280×1024 px). Once a sequence of two light pulses was recorded, the images were divided into small subsections which were cross-correlated with each other using flow map software (Flow Manager 4.4. Dantec Dynamics A/S, Skovlunde, Denmark). The correlation was achieved using an interrogation area of 32×32 pixels, allowing us to obtain valid measurements down to a particle displacement of 0.1 pixels. Using the equation,
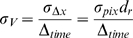
(1)with σ_Δ*x*_ the minimal displacement measurable (m), Δ*_time_* = 33 ms, the time separating two image record and *d_r_* = 27 µm the spatial resolution, one obtains the lowest detectable speed of 0.082 mm/s, and of 5.4 mm/s for a time interval of 500 µs. Conversely, with the maximal measurable particle displacement of 32 pixels, the maximal detectable speed is 2.62 cm/s for a time interval of 33 ms, and 17.3 cm/s for a time interval of 500 µs.

### Estimation of spider's velocity and profile extraction

*Pardosa* (Koch) is the most speciose genus among Holarctic wolf spider genera. Several species groups have been recognized, based on characteristics of the copulatory organs [31]. Based upon identification of mature males from our collecting sites, *Pardosa lugubris* (Walkenaer) was the most common species. However, this species was recently shown to incorporate distinct cryptic species whose immature individuals are, to date, impossible to differentiate (Kronestedt 2007). In our experiments, we used only immature spiders because they naturally spend much of their time hunting for prey and not seeking for partners. The mean body size was 3.6 mm (S.D. = 0.2 mm, N = 6). The body size was obtained by measuring the largest width of the prothorax, to which we added the lengths of coxa and the trochanter, as these three body parts act aerodynamically as a single unit. In the studied spiders, this unit was wider than the abdomen. During a single time interval of 33 ms, a spider travelled a distance of 5 mm when moving at a speed of 15 cm/s. There are therefore no data available on flow velocity for the 5 mm space next to the body surface. The distance from the body for which no information was available was greater for greater speeds.

In the horizontal set-up, we took care that the laser light sheet is focused 3 mm above the floor, at mid-height of the spider, just below the bottom eye row level. However, we cannot ascertain that the laser light sheet, which is diverging with an angle of 24° from the focal point, did not affect the spider, or during the low phase of the body oscillations. Whatever the amount of light spiders did get, it was much below the intensity of the bulk of the laser sheet, as we would otherwise see the eyes within the light sheet. We observed a tendency to avoid the laser light sheet rapidly in the vertical set-up.

We recorded 14 runs made by six *Pardosa* [*lugubris*] sp. spiders with the horizontal set-up. Measurements were only made when the spider velocity was assumed constant for several centimetres and spiders were running straight. The constant velocity assumption is derived from the measurements in [10] reporting an acceleration phase restricted to one centimetre, followed by a constant velocity. We therefore positioned the field of view of the camera at least 2–3 centimetres away from the entrance of the tunnel. The spider's velocity was determined by measuring the average velocity of the spider's body on a run. A run was restricted to the pairs of images (varying from one to five pairs) for which the images were of quality high enough for a faithful quantification of air flow. We extracted velocity profiles from the vector fields for each measurement. Profiles were evaluated along the upstream axis. In order to describe the flow velocity as faithfully as possible, we fitted the data with a flexible statistical function:
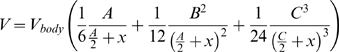
(2)With x being the distance to the spider's body (m), A = 0.0007, B = −0.0011 and C = 0.0179 and V_body_, the spider's body velocity (0.0944 m/s).
